# Foreign

**Published:** 1841-12-04

**Authors:** 


					﻿FOREIGN.
Letter on the Application of Soap to Burns. By
Thomas Williamson, of Edinburgh.—Consi-
dering the vast number of accidents which
daily occur by burning, it may not perhaps be
unacceptable to the profession to have their
attention directed to an application which I
have been in the habit of using in such cases
for some time past, with very decided advant-
age. The plan of treatment which I adopt,
and here allude to, is nothing novel, and may,
perhaps, be employed by many, if not known
to most. It is, therefore, solely with the view
of detailing what appears to me a most valua-
ble form of application, in cases of burning,
as well as of inviting its more general use,
(seeing that, in this neigbourhood at any rate,
it is not generally employed,) that 1 now trou-
ble you with these remarks. The more com-
mon of the applications generally employed,
appears to me liable to many objections. The
common sweet or train oil is a remedy fre-
quently resorted to, in the hurry and bustle of
the moment attending these accidents, by
friends or neighbours of the patient, who may
happen to be present at the time. This appli-
cation, too, is not unfrequently sanctioned by
the practitioner. Granting the occasional ul-
timate benefit of this application, and waving
its filthy nature, the strong objection to its use
is this—that it seldom or never affords instant
relief to the suffering patient. The same, I
think, holds true in respect of the linimentum
aquae calcis, or Carron oil, so very frequently
employed. The common raw cotton, or wad-
ding, has, of late years, and perhaps deserved-
ly too, held especial favour as a local applica-
tion. It must, however, be admitted, that the
patient derives no immediate relief to his suffer-
ings upon its first application; but, on the con-
trary, not unfrequently complains of augment-
ed pain for some time afterwards. This may
very easily be accounted for, both by its act-
ing as an irritating body, by its direct contact
with the red or raw surface, as well as by in-
creasing for a time the temperature of the part
to which it may be applied. Besides, it in a
few days becomes saturated with discharge
from the secreting surface, and consequently,
not only proves annoying to the patient, but
also a means of keeping up discharge in more
trivial cases. These objections are in a great
measure got rid of by using common soap,
which, besides its great value as a local appli-
cation, commands the ad litional advantage of
always being at hand in cases of emergency.
The mode in which I am in the habit of em-
ploying it is this:—A. common shaving box
may always be procured, from which a good
lather may, in the course of a minute or tv<m,
be easily obtained. This lather is then gently
laid over the burnt surface by means of a shav-
ing brush, and repeated so soon as the first
coat begins to dry, or the pain returns. This
practice ought to be repeated occasionally dur-
ing the first day, or until such time as the pain
is relieved. The benefit accruing to the pa-
tient is immediate, and the result of the prac-
tice highly satisfactory ; for in more superfi-
cial burns, if early applied, vesication is pre-
vented, and, in the course of a few days, des-
quamation of the cuticle follows, without leav-
ing a raw surface. Of course this, as a reme-
dial measure, is most applicable to superficial
burns ; but even in such cases as involve de-
struction of the more deep tissues, it is not used
without advantage, in so far as the personal
comfort of the patient is concerned. In such
cases, after the lapse of a few days, the erust
formed by the soap is easily removed, so as to
permit the employment of other remedies, if
necessary. I am not prepared to say whether
the benefit and instantaneous relief, following
the application of the lather, are to be ascribed
to its chemical composition, or simply to the
fact of its affording some degree of protection
from atmospheric agency, or both. Were
it necessary, I could easily adduce many cases
in illustration of its extreme value as a local
application; but as this would be needlessly
encroaching upon the space of your journal, I
leave it to those of the profession who have not
yet tested its efficacy, to substantiate, by the
result of their own experience, the truth of the
preceding statements.
London Medical Gazette.
On the advantage of keeping the Umbilical
Cord whole for some short time after birth. By
M. Baudelocque.—In all cases where the
infant is born weakly or in a state of asphyxia,
M. Baudelocque recommends not to cut the
umbilical cord for some time at least after
birth. He relates that since he has followed
the opinions of Smellie, Levret, Chaussier,
&c. on this subject, he has not lost a single
case, although when born the child might be
in a state of pretty complete asphyxia or apo-
plexy. He states that, though the child be
born in an apoplectic or asphyxiated state, the
circulation still continues through the umbili-
cal vein, even though the umbilical arteries
should have ceased to beat, and that premature
section of the umbilical cord takes away one
of the chief aids to its revival.—Ed. Med. and
Surg. Journ.,from Comptes Rendus de I'Acade-
mic des Sciences, 8th March, 1841.
We extract, entire, the following paper from
the Edinburgh Medical and Surgical Journal
for October, notwithstanding its length, be-
cause it forms a valuable monographic review
of a rare but most important class of accidents.
It is impossible to add the figures appended to
the original paper, nor would they be of much
avail; for, the language of the writer conveys
much clearer ideas than the badly executed
plate.
Case of perfect Ankylosis of the five Superior
Cervical Vert ebrae to each other, and Complete
Dislocation backwards of the 5 th from the 6/h,
without fracture. By Stephen S. Stanley,
Member of the Royal College of Surgeons in
London, and Assistant Surgeon of the Royal
Hospital, Haslar.— History of the case sent
with the patient; with the subsequent symp-
toms and treatment pursued by Dr. Mortimer,
the senior surgeon under whose care he was
placed.
George Weldon, aged 37, seaman, lost his
footing yesterday evening, the 20th of July,
1838, about nine o’clock, and fell backwards
on his head on the deck. I found him imme-
diately afterwards complaining of a severe
pain in the back part of his neck and between
the shoulders, and of pain and numbness in the
arms. His face was pale, and his pulse weak.
Five grains of Carb. Jlmmoniae in an ounce of
camphor mixture were administered, after
which he rallied. He is worse this morning,
complaining of numbness, not only in the arms,
but also in the legs; of the pain in the back
part of the neck being more severe, and of in-
ability to turn or move in any direction. As
the ship is in dock, it is thought advisable to
send him to Haslar Hospital for the benefit of
hospital treatment. Signed James M. Deas,
Assistant Surgeon, H. M. S. Pique, July 21,
1838.
Admitted into the Haslar Royal Naval Hos-
pital, on the 21st of July, 1838, at eleven A.M.,
in a state of perfect consciousness, without
wound or external appearance of bruise. Both
arms are commencingly paralytic, the left the
most so. The accident occurred yesterday
evening, since which he has not passed urine,
nor have the bowels been opened ; the pulse is
slow, weak and oppressed; pupils unaffected ;
nor does he refer to any complaint, save about
the muscles of the neck and shoulders.
His breathing is undisturbed. The catheter
was introduced ; a draught of infusion of senna
was immediately given, and an enema was ad-
ministered. Evening, a free evacuation from
the bowels had taken place; but the pulse was
quick and sharp. The enema was repeated;
and twenty ounces of blood were drawn from
the arm.
22d July—Respiration is hurried; the pulse
is weak; there are continual attempts to ex-
pectorate a frothy mucus, but the attempt is
ineffectual; was most anxious to inhale air;
he desired to be raised higher in bed; this is
accomplished with facility, by the rude yet ex-
cellent apparatus of Borthwick.
The enema was repeated; the urine was
withdrawn by a catheter; and six grains of
calomel and one scruple of jalap were given in
the form of bolus.
Noon—He is easier, he breathes more un-
interruptedly but the pulse flags. Evenino-_
Respiration laborious; death is approaching.
Died at half-past four o’clock on the morning
of the 23d, exactly fifty-five hours and a-half
after the accident, and forty-one hours and a-
half after his admission into this hospital.
Post-mortem.—On the posterior surface of
the body, extending from the occiput to as far
as the sixth or seventh dorsal- vertebra, there
was considerable ecchymosis ; and in making
a section of the integuments and subcutaneous
cellular tissue, a quantity of blood was found
effused into its texture. In prosecuting the
dissection further, especially in a space reach-
ing from the first cervical to the second dorsal
vertebra, coagulated blood in great quantity
was found surrounding the muscular fibres, a
number of which were ruptured and softened.
These being sponged away, a little more care-
ful dissection exposed to view a considerable
displacement backwards, of the fifth from the
sixth cervical vertebra. All the blood was
sponged and cleared away, and as much of the
soft parts removed as was possible, for the
purpose of ascertaining the exact position of
the dislocated vertebra. It was then found that
the little finger could easily be passed under-
neath it, into the spinal canal; and that the
body of the fifth pressed severely on the spinal
chord, and rested on the laminae and spinous
process of the sixth cervical vertebra. The
The spinal column was now removed (sawino-
through the angles of the ribs) at the seventh
dorsal vertebra. It was then ascertained be-
yond all doubt, that the injury was a complete
dislocation without fracture. The ligaments
and intervertebral substance were all ruptured;
and when suspended from above, the parts
were held together by the vertebral arteries and
spinal marrow, with its theca alone, the theca
vertebralis being uninjured.
Head.—The cranium was thick and very
heavy; the thinnest part measuring two lines
and a-half, and the thickest five lines and a-
half. The great longitudinal sinus was gorged
with blood, and so large as to admit with ease
the forefinger. The medullary substance of
the brain was soft and very vascular. When
the section of the centrum magnum ovale was
made, it was found studded with spots of red
blood. No other abnormal appearance was
observed until an attempt was made to remove
it, when it was found impossible to pass a knife
through the foramen magnum, to make a sec-
tion of the medulla oblongata. The brain was
however, removed, and it was then ascertained
that the foramen magnum, was so much con-
tracted, as scarcely to admit the point of the
little finger.
On closer inspection, and after dissecting off
the dura mater in this situation, the constric-
tion was evidently produced by the odontoid
process of the abscess being much larger than
natural, and projecting in a conspicuos manner
upwards towards the base of the brain, and
backwards on the medulla oblongata, which,
from the little that was attached to the pons
Varolii, appeared small and nearly flat. By
applying the saw at the posterior margin of the
foramen magnum, and carrying it obliquely
forwards and upwards, a section of the base of
the cranium was now made for the purpose of
ascertaining the exact condition of the odontoid
process, and the beautifully arranged ligaments
in this situation. The section being completed,
and a little dissection made, it was ascertained
that the whole of the cervical vertebrae, from
the atlas down to the seat of dislocation, were
completely ankylosed. Not the least vestige
of ligamentous structure could be observed,
with the exception of the capsular and occipito-
atlantal ligaments, forming the articulation be-
tween the occiput and atlas; and of these, the
capsular ligaments and synovial membranes,
when cut into, were found to be so much thick-
ened and altered in structure, as more to resem-
ble cartilage than ligament, and calculated to
impede seriously, if not altogether, the nodding
actions of the head and slight lateral motion,
which this articulation permits. No trace
could be found whatever of the. apparatus liga-
mentosus, and lateral ligaments, connecting the
occiput with the atlas; neither was there any
thing remaining in the form of the ligaments
which complete the articulation between the
atlas and axis; but nature, ever bountiful, had
formed a beautiful provision for the absence of
the transverse ligament by an isthmus of bone,
extending from the anterior aspect of the odon-
toid process to the posterior concave surface of
the anterior arch of the atlas; thus, in most re-
spects, answering every purpose for which the
transverse ligament is known, although placed
in a situation diametrically opposite.
After the usual process of maceration the
bones appeared white, and exceedingly com-
pact in their tissue, and, with the exception
of their ankylosed condition, are perfectly
normal:—their form in every respect not ap-
pearing to differ from the general characters
by which these vertebra are known.
The most remarkable feature in the whole
preparation, and evidently the result of a
former dislocation forwards, is the position of
the atlas; which, on the right side espe-
cially, is pushed forwards and upwards from
off the articulating surface of the axis, so as
to cause the odontoid process to present itself
nearly in the centre of the circle of the atlas.
A bridge of bone exactly half an inch in length,
and varying from three to lour lines in breadth,
passes nearly horizontally forwards, from the
odontoid process to the atlas, as described
above, and connects them together. The axis
is also pushed forwards in the same manner
from the third cervical vertebr,a but not to so
great an extent, giving the entire preparation a
twisted appearance to the left side. Its length,
measuring anteriorly, from the superior mar-
gin of the ring of the atlas to the inferior mar-
gin of the body of the fifth cervical vertebra,
is three inches and a half. The diameter of
the spinal foramen of the atlas, from behind
forwards, is exactly one inch and four lines,
and the transverse diameter one inch and half
a line. The odontoid process, instead of ter-
minating at its apex in a point, as it generally
dees, presents a broad and irregular ovoid
form, measuring transversely half an inch, and
from behind forwards, including the bony
bridge alluded to, one inch : its length is three-
fourths of an inch, and its distance from the
posterior arch of the ring of the atlas only four
lines.
Remarks.—It may be supposed that, having
ascertained the exact nature of this accident,
the author of this paper was very anxious to
obtain every particular relating to this man’s
history ; and as the Pique was still riding at
Spithead, he took the earliest opportunity of
going on board, for the express purpose of
gaining all the information possible; and he
has to thank Mr. Deas, the Assistant Sur-
geon, for his kindness in furthering his views
on that occasion.
It appears that the man had, for some years
past, always been subject to a stiff-neck, that
lie very often complained of rheumatic pains
in that region, and of sore throat. He was,
nevertheless, a very efficient and active sea-
man, alwaysjloing his duty, and never on the
sick list; but was unable to move his head to
one side, and was compelled to turn his whole
body round when he was desirous of looking
either to the right or to the left. It further ap-
pears that, at the time the time the accident
occurred, the deceased, although not drunk,
was in the state that sailors call “ rather
fresh,” and was “larking” with some of his
messmates, and in attempting to catch one of
them, his foot slipped, and he fell backwards,
his head only slightly striking the deck.
Complete dislocation of the vertebrae, with-
out frapture, (if the first and second cervical be
excepted,) is so very rare, that the occurrence
of such an accident is doubted, and even posi-
tively denied, by some of our best surgeons.
It is very desirable, therefore, that well au-
thenticated cases should be published. Sir
Astley Cooper, in his work on dislocations,
appears to doubt the possibility of a complete
dislocation, without fracture, though he does
not deny that such an accident may occur.
Delpech, Boyer, and some othdrs, assert that
it is utterly impossible ; and the latter gentle-
man gives some very sound anatomical facts,
in regard to the construction of the articula-
tions, for his reason in disbelieving it.
There are, however, several undoubted cases
on record; and Mr. Lawrence, Surgeon of
Bartholomew’s Hospital, has published a case,
if the author’s memory is correct, in the. Me-
dico-Chirurgical Transactions that ought to
convince the most sceptical. Sir Charles
Bell has also published, in his work on Inju-
ries of the Spine and Thigh Bone, a case of
complete dislocation of the last dorsal from the?'
first lumbar vertebra, (in a child that was
knocked down by a stage-coach,) with entire
division of the spinal cord. This patient sur-
vived, and was taken from the hospital, and
died thirteen months afterwards from croup :
there was, however, a small portion of the
bone broken off.
Professor Rust has also recorded a case of
dislocation in the cervical region, which was
replaced by himself. The injury occurred in
consequence of a severe fall on the head. The
The neck was completely bent to one side,
the upper extremities paralysed, and attacks of
convulsions and hiccough supervened. Re-
placement was attempted, and succeeded. The
patient was made to sit on the ground, and the
head drawn straight upwards by an able as-
sistant,—and the cure was completed by the
local application of cold.
Ehrlich has recorded a most remarkable
case of dislocation of the atlas. A young
man, 16 years of age, fell in carrying a sack
of flour upstairs, and his head was forcibly
bent forwards by the burden. He was found
senseless; with livid countenance, prominent
eyes, tongue hanging out of the mouth, with
slow and interrupted respiration, and pulse
scarcely perceptible. The limbs were motion-
less, and apparently paralysed; urine and
fasces passing involuntarily ; the head was in-
clined to the right side, and had lost its firm-
ness, so that it fell by its own weight from
side to side, when unsupported. The articular
process of the second vertebra projected on the
left side. Ehrlich considered it a dislocation
with pressure on the spinal cord, and caused
extension of the head to be made, while he en-
deavoured to force back the atlas, and bring
the secqnd vertebra forwards.
After some effort, the replacement was ef-
fected with a snap: the head now became
steady, and the arms moved; but the patient
remained insensible, with dilated pupils. The
respiration and pulse were improved; and, on
the next day, consciousness returned. A good
deal of swelling of the neck and ecchymosis
came on, but soon subsided; and after ten or
twelve days he quite recovered.
In all the accounts, however, which the au-
thor has read, he notices that the direction of
the displacement, when resulting from acci-
dent, has been forwards and upwards, and not,
as in this case, backwards and downwards.
That the ankylosis of the cervical vertebrae,
above the seat of the injury, may not have had
some influence in producing this anomaly, is
left to the more matured judgment of the elder
members of the profession; though the writer
is certainly disposed to think that the direction
of the dislocation, and the accident also, may
be accounted for, in a great measure, by their
abnormal condition. It appears very doubtful
that the slight fall which this man got, could,
under ordinary circumstances, have produced
such extensive and fatal injury.
With regard to the ankylosis in this instance,
there are a number of cases on record very si-
milar, though not to so great an extent. Wyn-
perse and Boyer have both met with cases of
dislocation of the occiput upon the atlas, and
of the latter upon the second vertebrae, with
ankylosis. Daubenton mentions a very re-
markable case of this kind, where the second
vertebra of the neck was pushed so far back as
to leave only an interval of three lines between
the odontoid process and the posterior arch of
the atlas; the second vertebra, at the same
time, inclining towards the right. It certainly
appears surprising that serious and fatal symp-
toms should not supervene, when we consider
that the spinal marrow must inevitably be com-
pressed in a very great degree; and it is a
matter of astonishment, how a patient could
possibly live sufficiently long for ankylosis to
be completed.
In this case, related by Daubenton, the dis-
tance between the odontoid process and the
posterior arch of the atlas, is one line less than
in the case related above.
Professor Sandifort, of Leyden, has described
a case where there was displacement of the at-
las and axis, with ankylosis of these two bones
to each other, and to the occiput: he describes
also several others, chiefly of ankylosis, be-
tween the occiput and atlas.
There is one remarkable case recorded by
him, where the occiput, all the cervical, and
the two superior dorsal vertebra?, were anky-
losed; but the articulating processes and lami-
' nae only were implicated, the bodies of the
I vertebra? being still connected by their fibro-
cartilage.
We have also in the museum, at Haslar, a
specimen of entire ankylosis of the secoYid and
third cervical vertebrae to each other; but there
does not appear to have been any displace-
ment.
There is another preparation, showing the
commencement of ankylosis in the dorsal re-
gion, at the articulating processes.
It would appear, therefore, that a more ex-
tensive case of complete ankylosis than the one
now under consideration, has not yet been au-
thenticated and published. There can be no
doubt that the primary disease has been ulcer-
ation of the cartilages, spontaneous dislocation
of the first and second vertebrae following.
Professor Rust, of Vienna, has given the best,
if not the only account, of this disease,—first.
in the Saltzburg Medico-Chirurgical Journal,
in 1813, and afterwards, more elaborately, in
his work on Diseases of the Joints, published
in 1817.
The first symptoms of this disease, accord-
ing Jto him, are, pain in the neck, becoming
more severe at night, or in swallowing a large
mouthful, or drawing a deep breath. The pain
affects one side of the neck, especially when
the head is moved towards the shoulder; it ex-
tends from the larynx towards the nape, and
often to the scapula of the pained side.
No external alteration is perceptible; but
firm pressure on the region of the first and se-
cond vertebra) produces considerable pain, and
thus points out the seat of the disease. The
difficulty of swallowing, and breathing, and
hoarseness, increase, alternating with pain in
the neck, which seems to fix about the back of
the head, and becomes intolerable on moving
that part. The head sinks towards one shoul-
der, the face being turned a little down; for, in
general, the articulations are affected on one
side only, and that was the left, in seven out
of nine examinations, after death. If both
sides are affected, the head will incline directly
forwards. In this state, things continue for
several weeks or months; and, before worse
symptoms come on, there is often apparent im-
provement, freer motion, and more natural situ-
ation of the head. But the uneasiness in speak-
ing and swallowing returns; the pain becomes
more severe and extensive; the head falls a lit-
tle backwards, and sinks towards the opposite
side. The patient feels as if the head were too
heavy, and he carefully supports it with his
hands, when he moves from the sitting to the
lying position, or vice versa. This may be
considered a pathognomonic symptom of the
affection. Another symptom which, at this pe-
riod, shows the true nature of the disease, is a
peculiar expression of pain in the countenance;
which, combined with the position and stiffness
of the head, constitutes so characteristic an as-
semblage of appearances, that it is enough to
have seen it once in order to recognize it again
immediately. In the further progress of the
case, noise in the head, deafness, giddiness,
cramps and convulsions, partial paralysis, par-
ticularly of the upper limbs, loss of voice, pu-
rulent expectoration, and hectic . symptoms,
supervene. Generally, no external change is
observable, either in the neck or in the cervical
region; and Rust observed, in one case only,
swelling of the affected side, which broke, and
left fistulous ulcers.
But the slightest pressure in the region of
the three upper vertebrae is acutely painful;
and, sometimes, in the advanced period of the
disease, a grating of rough surfaces is distinct-
ly perceived, when the head is turned.
patient may continue for months in this help-
less pnd painful state, and then dies,, either
from exhaustion or debility; or, which is more
frequent, suddenly and unexpectedly. Expe-
rience has furnished very little satisfactory
knowledge in regard to the treatment of this
disease.
Blisters, setons, and issues have been tried;
but Rust found these remedies only capable of
retarding the progress of the disease, and pro-
ducing little or no abatement in the symptoms.
The only cure is that effected by nature ;
and the best treatment appears to be that,
where such a position, and quietude of the pa-
tient is adopted, which favours its terminating
in ankylosis.
It appears rather strange that the ankylosis
should have terminated so abruptly in this
case. There was no appearance whatever of
a disposition to change in the articulation be-
tween the fifth and sixth vertebra, the seat of
dislocation ; the ligaments in this situation
(although ruptured) were distinctly capable of
being made out, and those immediately suc-
ceeding are perfectly normal, with the excep-
tion of the ligamenta subfava, in the dorsal re-
gion, where there are evident symptoms of os-
sific change.
Interments in the City and Liberties of Phi-
ladelphia, from the 20th to the 21th of No-
vember.
> ci	p* a
a. =;	S'.
Diseases. — g:	Diseases.	E
. m	• rt
? ?
Asphyxia,	0	1 [Brought forward,26	30
Apoplexy	1	0 Mania a Potu,	3	0
Burns, "	0	1 Neglect,	1	0
Croup,	0	3 Old age,	2	0
Consumption of	Pleurisy,	1 0
the lungs,	11	2.Small pox,	3	6
Concussion of	;Still-born,	0 5
brain,	1	0 Suicide,	1	0
Convulsions,	0	5 Teething,	1	0
Cramp of Sto-	Unknown,	1	0
mach,	10	—	—
Diarrhoea,	1	0 Total,	80—38	42
Dropsy,	1	0
------ Head, 0 2	-----
Dysentery,	1	0
Debility	1 0 Of the above, there
Epilepsy,	0 1 were under 1 year, 20
Fever,intermittentO 1 From 1 to 2	4
----Nervous, 10:	2 to 5	12
	Typhus,	10	5	to	10	2
	Typhoid,	11	10-----to---15-0
--------------------------------------------------------------------------------------------------------------------Scarlet,	0	5	15	to	20	4
Inflammation, 1	0	20 to 30	12
_____ Brain,	1	1	30	to	40--11
-------------------------------------------Bronchi, 11--------------------------------40 to 50------------------------4
-------------------------------------------Lungs,-------------------------------------1------------------------------------3|----------------------------------50--------------------------------to------------------------------60----------------------------3
------------------------------------------- Stomach,----------------------------------0---------------------------------1--------------------------------60------------------------------to----------------------------70--------------------------1
-------------------------------------------Heart,	1	0	70	to	80	4
— Larynx	0	1	80	to	90	3
Marasmus,	0	1	—
--------------------Total,	80
Carriedforward, 26 30
				

